# Boosting the Hydrogen Evolution Activity of a Low‐Coordinated Co─N─C Catalyst via Vacancy Defect‐Mediated Alteration of the Intermediate Adsorption Configuration

**DOI:** 10.1002/advs.202415665

**Published:** 2025-01-13

**Authors:** Qianwei Song, Zhichao Gong, Jianbin Liu, Kang Huang, Gonglan Ye, Shuwen Niu, Huilong Fei

**Affiliations:** ^1^ State Key Laboratory for Chemo/Biosensing and Chemometrics Advanced Catalytic Engineering Research Center of the Ministry of Education and College of Chemistry and Chemical Engineering Hunan University Changsha 410082 P. R. China; ^2^ College of Chemistry and Chemical Engineering Institution Qingdao University Qingdao 266071 P. R. China

**Keywords:** adsorption configuration, hydrogen evolution reaction, low coordination, single‐atom catalysts, vacancy defects

## Abstract

The cobalt‐nitrogen‐carbon (Co─N─C) single‐atom catalysts (SACs) are promising alternatives to precious metals for catalyzing the hydrogen evolution reaction (HER) and their activity is highly dependent on the coordination environments of the metal centers. Herein, a NaHCO_3_ etching strategy is developed to introduce abundant in‐plane pores within the carbon substrates that further enable the construction of low‐coordinated and asymmetric Co─N_3_ sites with nearby vacancy defects in a Co─N─C catalyst. This catalyst exhibits a high HER activity with an overpotential (*η*) of merely 78 mV to deliver a current density of 10 mA cm^−2^, a Tafel slope of 45.2 mV dec^−1^, and a turnover frequency of 1.67 s^−1^ (at *η* = 100 mV). Experimental investigations and theoretical calculations demonstrate that the vacancy defects neighboring the Co─N_3_ sites can modulate the electronic structure of the catalyst and alter the adsorption configuration of the H intermediate from the typical atop mode to the side mode, resulting in weakened H adsorption strength and thus improved HER activity. This work provides an efficient strategy to regulate the coordination environment of SACs for improved catalytic performance and sheds light on the atomic‐level understanding of the structure‐activity relationships.

## Graphical abstract

The introduction of vacancy defect near the low‐coordinated cobalt active sites leads to the weakened H intermediate adsorption and further improvement in the activity toward the hydrogen evolution reaction.

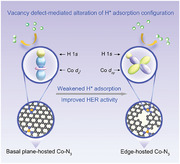

## 1 Introduction

The excessive consumption of fossil fuel and the associated concomitant environmental pollution call for the exploration of green renewable energy for mitigating greenhouse gas emissions.^[^ ^1^ ^]^ Hydrogen produced by electrocatalytic hydrogen evolution reaction (HER) from water is considered to be a promising clean energy carrier, particularly when utilizing solar and wind power as electricity sources.^[^ ^2^ ^]^ Hitherto, platinum (Pt) metals located at the top of the volcano plot are acknowledged as the most optimal electrocatalyst for H_2_ production according to the Sabatier principle.^[^ ^3^ ^]^ However, the low reserve and high cost of Pt hamper its large‐scale commercial application.^[^ ^4^ ^]^ Hence, it is urgently needed to develop highly efficient and durable electrocatalysts with earth‐abundant materials.

Atomically dispersed transition metal sites coordinated with nitrogen and embedded in carbon matrix (M─N─Cs) represent a unique class of single‐atom catalysts (SACs).^[^ ^5^ ^]^ Owing to the 100% atom utilization efficiency and the tunable coordination configuration, M─N─Cs are considered as one of the most promising candidates for electrocatalysis and they have been widely studied over the last decade.^[^ ^6^ ^]^ Generally, the catalytic performance of M─N─Cs depends on the inherent chemical and electronic states of the central metal atoms, where the optimal active sites exhibit moderate binding strength for intermediates.^[^ ^7^ ^]^ This is because that too strong binding causes intermediates hard to transform into products, whereas too weak binding results in low coverage with intermediates that even desorb before the products are formed.^[^ ^8^ ^]^ The prototypical active site configuration of M─N─Cs is MN_4_ moiety composed of central metal sites coordinated with four equivalent N ligands in planar *D*_4__h_ configuration.^[^ ^9^ ^]^ However, both experimental and theoretical investigations have indicated that such symmetric structure is not conducive to the further improvement of the catalytic activity as the symmetric charge distribution is incompetent to adsorb and activate intermediates.^[^ ^10^ ^]^ For example, Lu et al., observed that the nitrate electrochemical reduction reaction (NO_3_RR) rates were unfavorable due to the weak adsorption of NO_3_^−^ on the symmetric CuN_4_ configuration and that the adsorption could be improved by the enhanced polarity of Cu site coordinated with two N atoms and two O atoms.^[^ ^11^ ^]^ Liu et al., found that the symmetric electron distribution of the FeN_4_ site was undesirable for the adsorption and activation of O_2_ while the FeN_4_─O active sites with axial O coordinator achieved efficient oxygen reduction reaction (ORR) activity as a result of electron localization induced by charge transfer from FeN_4_ to axial O atom.^[^ ^12^ ^]^ Thus, broken‐symmetry atomic metal centers could endow M─N─Cs with desirable catalytic performance.^[^ ^13^ ^]^ Accordingly, various coordination engineering strategies, such as tuning the coordination number and the coordinator types of the active center, have been developed to construct the asymmetric configurations.^[^ ^14^ ^]^ Aside from the distorted structure arising from variations in the coordination environment, the geometric environment surrounding the active center can also influence the electron redistribution and optimize the intermediate binding energies.^[^ ^15^ ^]^ For example, it has been reported that the performance of the metal sites hosted at the edge of vacant sites or the perimeter of pores is superior to those in the basal plane because of the rearrangement of electronic structures.^[^ ^16^ ^]^ Additionally, the introduction of porous structure is also conducive to mass transport and exposure of active sites, thereby improving electrocatalytic activity.^[^ ^17^ ^]^ Though substantial progress has been made in the activity improvement for M─N─Cs, the existing methods generally focus on the separate regulation in either the coordination configuration or the geometric environment, while strategies combining these two effects to better tune the electronic structure of M─N─Cs have been underexplored.

Herein, we achieved the simultaneous regulation of coordination configuration and geometric environment via NaHCO_3_ etching to construct low‐coordinated Co─N_3_ sites preferentially hosted in the edge plane of porous graphene (denoted as Co─N_3_/EG), which demonstrated improved HER performance through regulated adsorption configuration of reaction intermediates. Various characterizations uncovered the modified electronic structure of the catalyst with varied oxidation states and spin states of the Co center. When applied as an electrocatalyst toward HER, Co─N_3_/EG exhibited outstanding activity in acidic media with overpotential (*η*) at 10 mA cm^−2^ as low as 78 mV, and a small Tafel slope of 45.2 mV dec^−1^, making it one of the most active catalysts among SACs. Density functional theory (DFT) calculations suggested that the hydrogen intermediate (H^*^) at the top position of active sites preferably shifted to the side mode when vacancy defect was introduced near the Co center, thus weakening the electronic coupling between the active site and intermediate that further led to enhanced HER activity.

## 2 Results and Discussion

### 2.1 Synthesis and Characterizations

**Figure 1:**
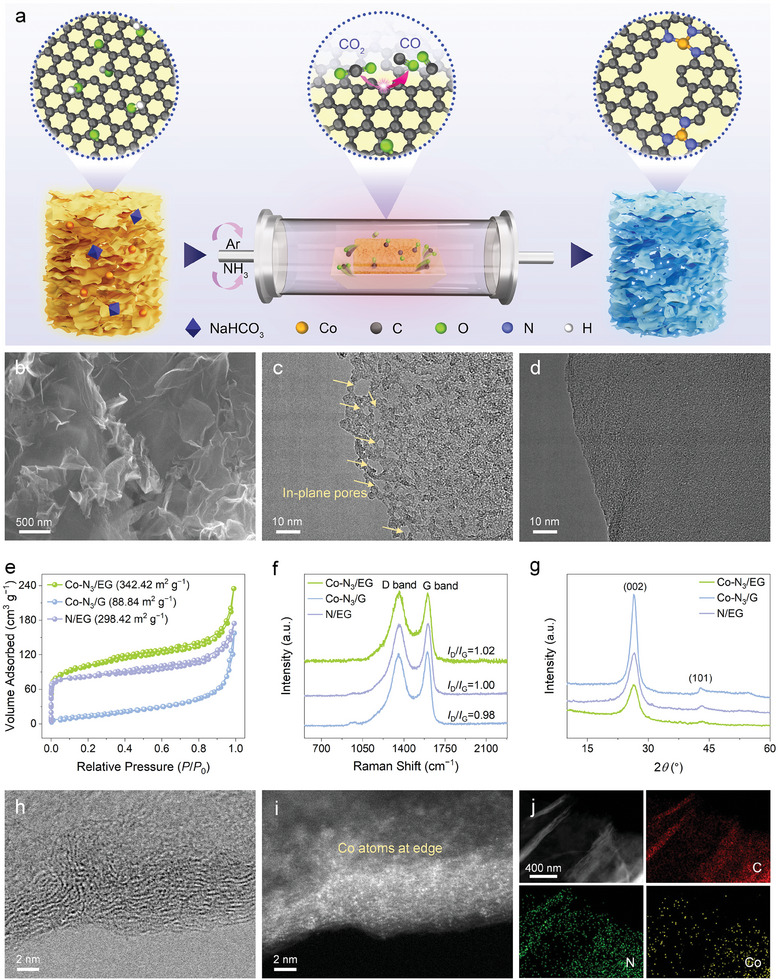
Synthesis and structural characterizations. a) Schematic illustration of the procedure for preparing Co─N_3_/EG. b) SEM image of Co─N_3_/EG. TEM images of c) Co─N_3_/EG and d) Co─N_3_/G. e) N_2_ adsorption‐desorption isotherms, f) Raman spectra, and g) XRD patterns of Co─N_3_/EG, Co─N_3_/G and N/EG. h) Bright‐field STEM image and the corresponding i) dark‐field STEM image of Co─N_3_/EG. j) STEM image and the corresponding elemental maps of Co─N_3_/EG.

As schematically illustrated in **Figure** **1**a, the preparation of Co─N_3_/EG was achieved via a simple impregnation‐calcination method with NaHCO_3_ as the pore‐forming agent. Briefly, graphene oxide (GO) was selected as the support to immobilize CoCl_2_ due to the electrostatic interaction between oxygen‐functional groups in GO and Co^2+^ ions. Then, the hybrid was mixed with NaHCO_3_ and dried by lyophilization. Finally, the dried sample in the form of 3D foam was subjected to thermal reduction under Ar/NH_3_ atmosphere accompanied by the release of CO_2_ by NaHCO_3_ decomposition. The CO_2_ served as an etchant at high temperatures to generate defects and porous structure within the graphene matrix via the Boudouard reaction (C + CO_2_ → 2CO).^[^ ^18^ ^]^ As control samples, a pore‐deficient Co─N─C catalyst (denoted as Co─N_3_/G) was prepared following the same procedure as Co─N_3_/EG except the addition of NaHCO_3_ was omitted, while a metal‐free catalyst (denoted as N/EG) was prepared without the addition of Co precursors. More detailed descriptions of the preparation procedures were provided in the Experimental Section.

Scanning electron microscopy (SEM) image showed that Co─N_3_/EG had a crumpled 2D flake‐like morphology (Figure 1b). Transmission electron microscopy (TEM) image revealed that there were abundant in‐plane pores with sizes ranging from 1 to 10 nm in the graphene substrate of Co─N_3_/EG (Figure 1c). Similar porous structure was observed in N/EG (Figure S1, Supporting Information). In contrast, Co─N_3_/G exhibited a more intact and smooth morphology without the obvious presence of pores in the graphene substrate (Figure 1d; Figure S2, Supporting Information). N_2_ adsorption‐desorption measurements were employed to analyze the Brunauer–Emmett–Teller (BET) surface area and pore size distribution of the samples (Figure 1e; Figure S3, Supporting Information). The isotherms of Co─N_3_/EG and N/EG exhibited a sharp rise at the low‐pressure region and a hysteresis loop at a higher pressure, suggesting a hierarchical micro/mesoporous structure. In comparison, these characteristics were absent in the isotherm profile of Co─N_3_/G, indicative of its inferior porosity. The quantitative analysis revealed that Co─N_3_/EG had the largest surface area of 342.42 m^2^ g^−1^, followed by N/EG of 298.42 m^2^ g^−1^ and Co─N_3_/G of 88.84 m^2^ g^−1^. In addition, the pore volumes of Co─N_3_/EG, N/EG and Co─N_3_/G were determined to be 0.56, 0.40, and 0.13 cm^3^ g^−1^, respectively. The increased surface area and pore volume of Co─N_3_/EG could enable the formation of edge‐hosted active sites as well as facilitate the mass transport and exposure of active sites. The defect‐rich nature of Co─N_3_/EG was further studied by Raman spectroscopy. As depicted in Figure 1f, two characteristic peaks were displayed at 1362 and 1602 cm^−1^ assigned to the disorder‐associated D band and graphitization‐related G band, respectively. The higher *I*_D_/*I*_G_ in Co─N_3_/EG and N/EG than that in Co─N_3_/G highlighted the abundance of edge defects introduced by high‐temperature CO_2_ etching. Powder X‐ray diffraction (XRD) patterns showed that the diffraction peaks of the (002) and (101) plane at ≈26.4° and ≈43.2° for Co─N_3_/EG and N/EG were much broader and weaker than those for Co─N_3_/G (Figure 1g), suggesting the poor crystallinity of graphitic carbon and higher structural disorder in Co─N_3_/EG and N/EG. In addition, no diffraction peak characteristic of metallic crystallites was detected in all samples. Bright‐field and dark‐field scanning transmission electron microscopy (STEM) images of Co─N_3_/EG displayed the concentrated distribution of atomic Co sites at the graphene edge (Figure 1h,i). For the control sample of Co─N_3_/G, the atomic Co species were mainly located in the basal plane due to the limited in‐plane holes (Figure S4, Supporting Information). Energy dispersive X‐ray spectroscopy (EDS) maps showed that the C, N, and Co elements were uniformly distributed in Co─N_3_/EG (Figure 1j).

### 2.2 Characterizations of Atomic and Electronic Structure

**Figure 2:**
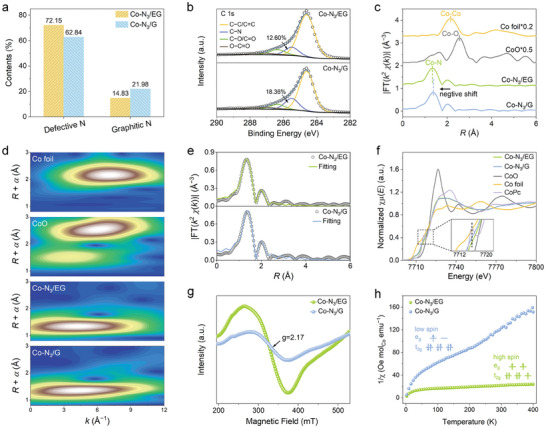
Characterizations of atomic and electronic structure. a) Relative contents of defective N (pyridinic and pyrrolic N) and graphitic N determined by N 1*s* XPS analysis. b) C 1*s* XPS spectra of Co─N_3_/EG and Co─N_3_/G. c) *k* ^2^‐weighted EXAFS−FT curves of Co─N_3_/EG, Co─N_3_/G and reference samples of bulk CoO and Co foil. d) EXAFS‐WT spectra of Co─N_3_/EG, Co─N_3_/G, and reference samples of bulk CoO and Co foil. e) EXAFS‐FT fitting curves of Co─N_3_/EG and Co─N_3_/G. f) Co *K*‐edge XANES spectra of Co─N_3_/EG, Co─N_3_/G, and reference samples of bulk CoO, Co foil, and CoPc. g) EPR spectra and h) M‐T curves of Co─N_3_/EG and Co─N_3_/G.

X‐ray photoelectron spectroscopy (XPS) survey spectra of Co─N_3_/EG, Co─N_3_/G, and N/EG showed the presence of C, N, and O elements and a weak peak ≈780 eV associated with Co was observed in the spectra of Co─N_3_/EG and Co─N_3_/G due to its low content (Figure S5, Supporting Information). The Co contents in Co─N_3_/EG and Co─N_3_/G were determined to be 1.33 and 1.30 wt.%, respectively, by inductively coupled plasma mass spectrometry (ICP‐MS). The compositions of all samples were summarized in Table S1 (Supporting Information). The high‐resolution Co 2*p* XPS spectra suggested that the Co was in an ionic state and additionally, the Co 2*p*_3/2_ peak for Co─N_3_/EG showed a slight positive shift of ≈0.45 eV relative to that of Co─N_3_/G, implying the decreased electron density of the Co center (Figure S6, Supporting Information). For the high‐resolution N 1*s* XPS spectra (Figure S7, Supporting Information), the N species can be deconvoluted into four types, including pyridinic N (398.4 eV), pyrrolic N (399.7 eV), graphitic N (401.3 eV), and oxidized N (402.7 eV). Quantitative analysis elucidated that the population of the defective N species (including the pyridinic and pyrrolic N) was higher in Co─N_3_/EG than Co─N_3_/G (**Figure** **2**a), which could be ascribed to the higher porosity and enriched edge sites in Co─N_3_/EG. This observation was further corroborated by the high‐resolution C 1*s* XPS spectra that showed a lower relative content of C─N species in Co─N_3_/EG (12.60%) than Co─N_3_/G (18.36%) (Figure 2b), considering that the defective N located at the edge of the carbon matrix bonds with two adjacent carbon atoms while the graphitic N embedded in basal plane bonds with three carbon atoms.^[^ ^19^ ^]^ Next, extended X‐ray absorption fine structure (EXAFS) and X‐ray absorption near‐edge structure (XANES) measurements were conducted to investigate the coordination configuration and electronic structure of Co─N_3_/EG and Co─N_3_/G. The EXAFS Fourier transform (EXAFS‐FT) spectra disclosed a prominent peak at ≈1.34 Å for Co─N_3_/EG and Co─N_3_/G (Figure 2c), which can be ascribed to the Co─N coordination in the first coordination shell. Careful observation revealed that the major peak of Co─N_3_/EG shifted slightly toward a lower *R*‐value compared to that of Co─N_3_/G, suggesting the shortened Co─N bond in Co─N_3_/EG. No peaks related to Co─Co bonds, as seen in the Co foil reference, were observed in Co─N_3_/EG and Co─N_3_/G. The EXAFS wavelet transform (EXAFS‐WT) spectra detected only one intensity maximum at ≈4.21 Å^−1^ (Figure 2d), confirming the atomic dispersion of the Co species in Co─N_3_/EG and Co─N_3_/G. To further determine the Co─N bond lengths and coordination structure, quantitative least‐squares EXAFS curve‐fitting analysis was performed (Figure 2e; Figure S8, Supporting Information). The fitting results indicated that Co─N_3_/EG had a contracted interatomic distance (1.87 Å) for the Co─N bond compared with Co─N_3_/G (1.91 Å) (Table S2, Supporting Information). In contrast to the conventional symmetric Co─N_4_ configuration, the coordination number in the first coordination sphere in Co─N_3_/EG and Co─N_3_/G were estimated to be 2.8 and 3.0, respectively, indicating the adoption of the low‐coordinated Co─N_3_ moiety.

XANES spectra uncovered that the Co *K*‐edge absorption of Co─N_3_/EG and Co─N_3_/G were situated between the Co foil and CoO (Figure 2f), signifying the oxidation state of Co was between 0 and +2. The more positive absorption edge of Co─N_3_/EG suggested that the Co single atoms in the edge‐hosted Co─N_3_ sites were oxidized to a larger extent than those in the basal‐plane‐hosted counterparts in Co─N_3_/G, in line with XPS results. In addition, the pre‐edge 1*s*→4*p*_z_ transition peak at ≈7715 eV, which was a well‐established fingerprint to a square‐planar configuration of the Co─N_4_ moiety with high *D*_4__h_ symmetry as observed in Co phthalocyanine (CoPc), was largely suppressed in Co─N_3_/EG, indicating its broken symmetric configuration. Electron paramagnetic resonance (EPR) and zero‐field cooling (ZFC) temperature‐dependent magnetic susceptibility (M‐T) were conducted to study the electron spin state. In EPR spectra (Figure 2g), the characteristic signal at *g*‐value of 2.17 was attributed to the unpaired electrons in the Co *d* orbitals, and the enhanced EPR signal in Co─N_3_/EG proved the increment of unpaired electrons compared with Co─N_3_/G. For the M‐T curves (Figure 2h), the values of effective moments were 5.05 *μ*_B_ for Co─N_3_/EG and 3.04 *μ*_B_ for Co─N_3_/G by fitting the M‐T curves based on the Langevin equation.^[^ ^20^ ^]^ The number of unpaired *d*‐orbital electrons on the Co center was calculated via the equation of $\mu_{\mathrm{eff}}=\sqrt{n(n+2)}$. Accordingly, the number of unpaired *d* electrons of Co was 3.33 and 1.41 for Co─N_3_/EG and Co─N_3_/G, respectively, suggesting that the Co ions in Co─N_3_/EG adopted the high‐spin states.

### 2.3 Electrocatalytic Performance Toward HER

**Figure 3:**
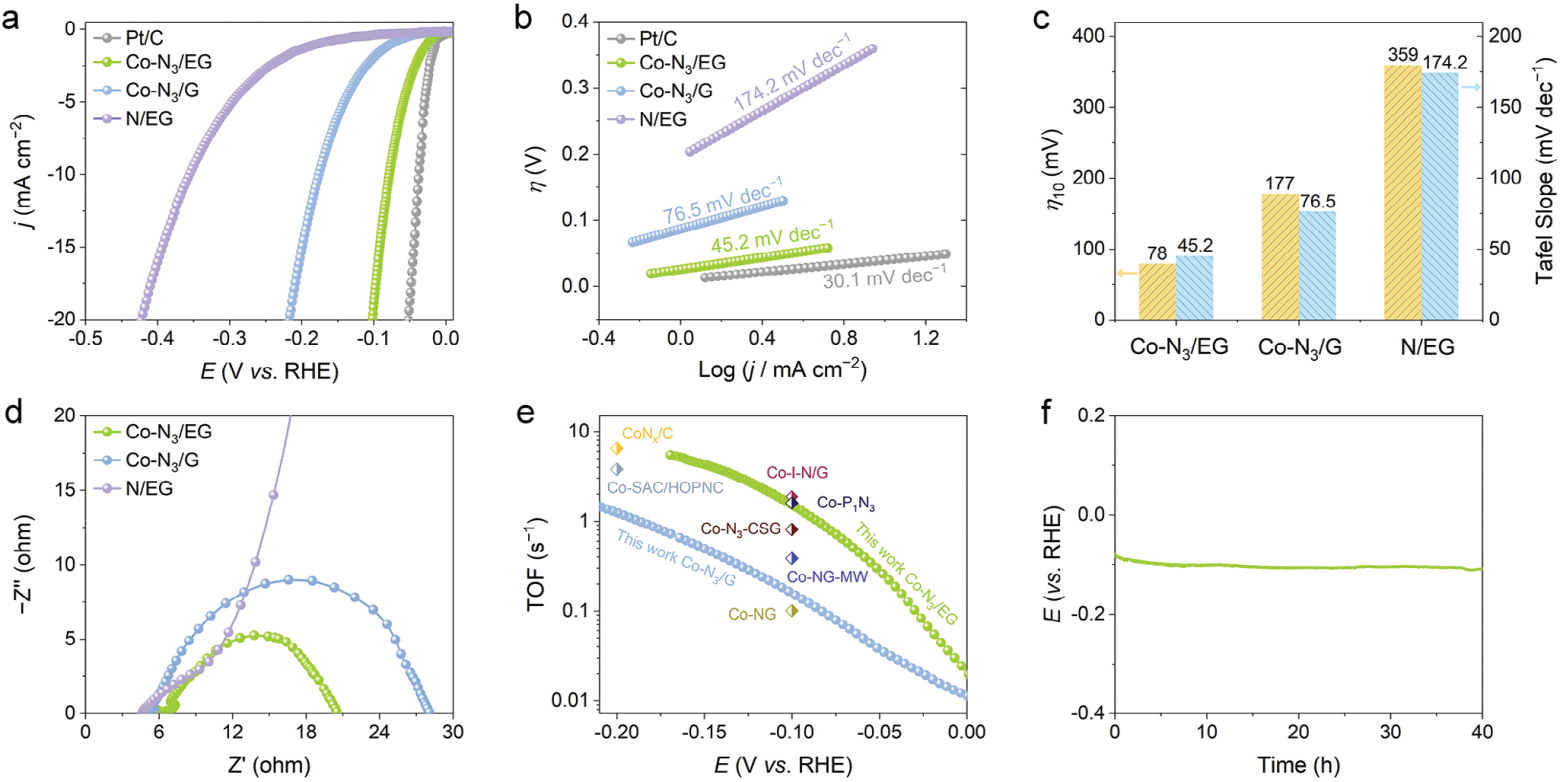
Electrocatalytic HER performance. a) LSV curves of Co─N_3_/EG, Co─N_3_/G, N/EG and 20 wt.% Pt/C. b) Tafel plots derived from the LSV curves of the corresponding samples in (a). c) The comparison between Co─N_3_/EG and Co─N_3_/G in terms of *η* at 10 mA cm^−2^ and Tafel slopes. d) EIS spectra of Co─N_3_/EG, Co─N_3_/G and N/EG. e) TOF values of Co─N_3_/EG and Co─N_3_/G compared with recently reported Co‐based SACs (Table S3, Supporting Information). f) Stability evaluation of Co─N_3_/EG by chronopotentiometric test at a constant current density of 10 mA cm^−2^ for 40 h.

The electrocatalytic HER activity of the materials was assessed in 0.5 m H_2_SO_4_ electrolyte with a typical three‐electrode system. The reference electrode of Hg/Hg_2_SO_4_ was calibrated against a reversible hydrogen electrode (RHE) prior to test (Figure S9, Supporting Information). A series of experimental conditions, including the carbonate species, the NaHCO_3_ content, and the first‐step and second‐step annealing temperatures, were also varied to optimize the catalytic activity (Figures S10 and S11, Supporting Information). The linear sweep voltammetry (LSV) curves of the optimal Co─N_3_/EG, Co─N_3_/G, and N/EG, along with the commercial 20 wt.% Pt/C as reference, were collected in **Figure** **3**a and the corresponding Tafel plots were displayed in Figure 3b. It was obvious that Co─N_3_/EG revealed a greatly enhanced HER activity over Co─N_3_/G and N/EG. Specifically, Co─N_3_/EG possessed an onset *η* as low as ≈17 mV (defined at the current density of 1 mA cm^−2^) and it required a small *η* of 78 mV to reach the benchmark current density of 10 mA cm^−2^, which was much lower than Co─N_3_/G (177 mV) and N/EG (359 mV). Notably, compared to the previously reported Co‐based SACs, Co─N_3_/EG was among the most active HER catalysts (Table S3, Supporting Information). The Tafel slope of Co─N_3_/EG was 45.2 mV dec^−1^, which was much smaller than Co─N_3_/G (76.5 mV) and N/EG (174.2 mV dec^−1^) (Figure 3c), indicating its superior reaction kinetics. The enhanced reaction kinetics of Co─N_3_/EG was further confirmed by the electrochemical impedance spectroscopy (EIS) measurements as the Nyquist curves revealed that Co─N_3_/EG had the smallest charge transfer resistance (Figure 3d). The activity of Co─N_3_/EG experienced a notable decrease upon the addition of poison reagent of KSCN (Figure S12, Supporting Information), suggesting that the main active sites were the Co moieties. The electrochemical active surface areas (ECSA) of Co─N_3_/EG, Co─N_3_/G, and N/EG were determined from the double‐layer capacitance measured in the non‐Faradaic potential region (Figure S13, Supporting Information). The ECSA of Co─N_3_/EG (189.08 m^2^ g^−1^) and N/EG (117.95 m^2^ g^−1^) were significantly higher than Co─N_3_/G (45.13 m^2^ g^−1^). The high ECSA value of Co─N_3_/EG was beneficial in facilitating the active site exposure and mass transfer. Upon normalizing the polarization curves by ECSA, the specific activity of Co─N_3_/EG was still higher than that of Co─N_3_/G (Figure S14, Supporting Information), suggesting that the enhanced performance of Co─N_3_/EG arose not only from the increase in active surface area but also from an enhancement in intrinsic activity. Furthermore, to compare the intrinsic activity of Co─N_3_/EG and Co─N_3_/G, their turnover frequency (TOF) was calculated with the assumption that each Co site contributed to one active site. As shown in Figure 3e, the TOF values of Co─N_3_/EG were superior to those of Co─N_3_/G at all potentials. For example, the TOF value of Co─N_3_/EG at *η* = 100 mV was 1.67 s^−1^ which was ≈2 times as high as that of Co─N_3_/G (0.80 s^−1^). Significantly, the TOF values of Co─N_3_/EG were superior to most of the previously reported Co‐based SACs (Table S3, Supporting Information). Moreover, the cyclic voltammetry (CV) sweeping test up to 4000 cycles and the 40−h chronopotentiometric test at a constant current density of 10 mA cm^−2^ indicated the excellent catalytic stability of Co─N_3_/EG (Figure 3f; Figure S15, Supporting Information). The XPS spectra and dark‐field STEM image of Co─N_3_/EG collected after the durability test suggested that Co remained in the ionic state and its atomic dispersion was maintained (Figures S16 and S17, Supporting Information).

### 2.4 Theoretical Investigation of HER Activity via DFT Analysis

**Figure 4:**
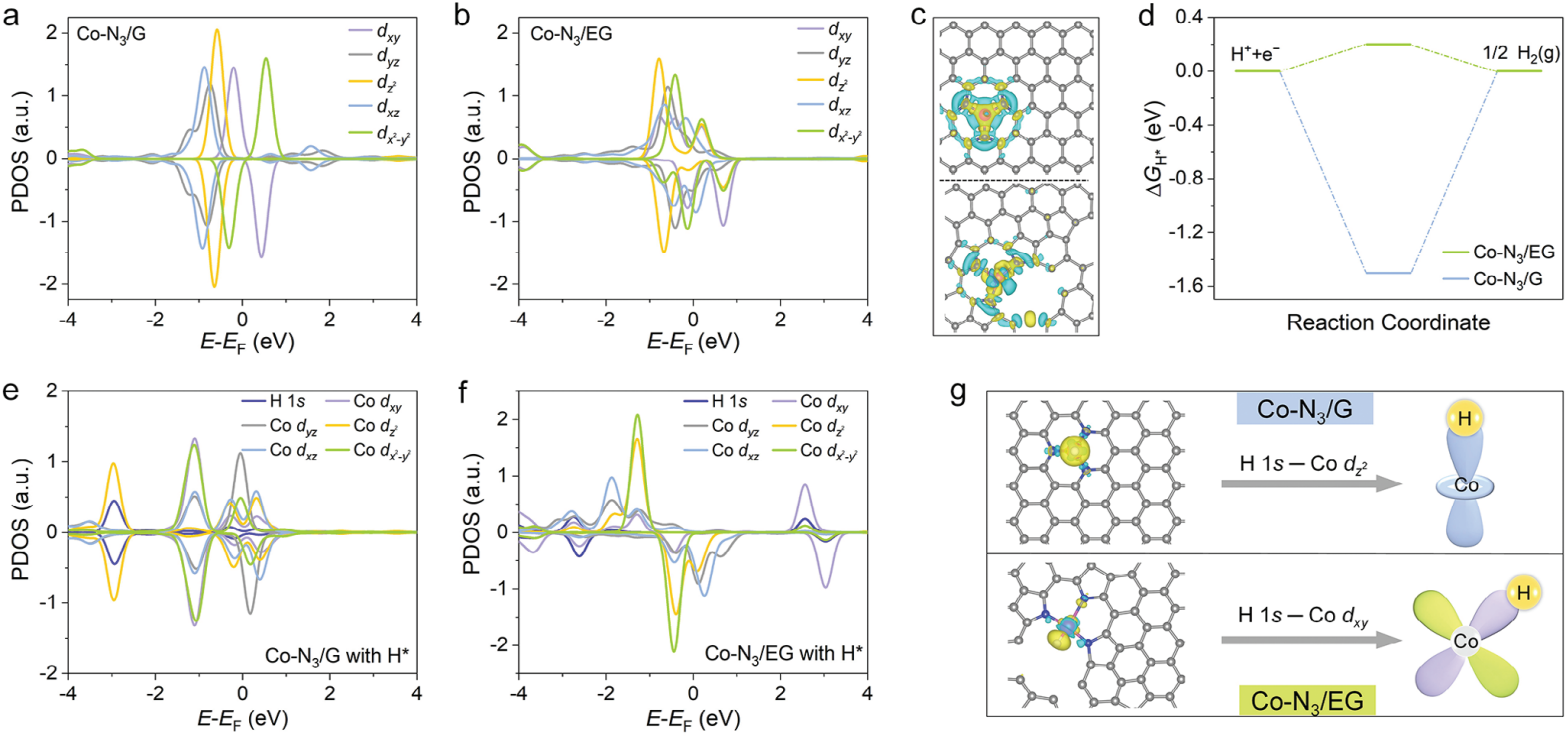
Theoretical investigation. PDOS diagrams of a) Co─N_3_/G and b) Co─N_3_/EG. c) Charge density differences of Co─N_3_/EG (bottom) and Co─N_3_/G (top). Yellow and cyan isosurfaces are represented as charge density accumulation and depletion areas, respectively. d) Gibbs free‐energy diagrams of HER on Co─N_3_/EG and Co─N_3_/G. The PDOS diagrams of e) Co─N_3_/G and f) Co─N_3_/EG with adsorbed H intermediate. g) Charge density differences of Co─N_3_/EG (bottom) and Co─N_3_/G (top) with adsorbed H intermediate and the corresponding schematic diagram for the orbital interaction between the Co sites and the intermediate.

To gain insights into the origin of the enhanced HER activity on Co─N_3_/EG, DFT calculations were carried out. The structural models of edged‐hosted Co─N_3_ motif in Co─N_3_/EG and in‐plane Co─N_3_ motif in Co─N_3_/G were first constructed and optimized based on the structural information acquired in experiments (Figure S18, Supporting Information). The projected density of states (PDOS) analysis was first performed to better probe the influence of the micro‐environment on the electronic structure of the Co sites (**Figure** **4**a,b). Compared with Co─N_3_/G, an increase in the spin state of Co *d_x_* ^2^_‐_ *_y_* ^2^ orbital below the Fermi level was observed in Co─N_3_/EG, suggesting that the electrons were transferred from the lower orbital to the higher orbital. In other words, the edge occupancy of the Co─N_3_ site contributed to the rearrangement of the 3*d* orbital electrons and caused the high spin polarization in Co─N_3_/EG. Additionally, the total DOS analysis indicated that there was an increase in the local density of states at the Fermi level for Co─N_3_/EG compared to Co─N_3_/G (Figure S19, Supporting Information). The differential charge density and Bader charge analysis revealed the electron transferred from the Co atom to the coordinated N atoms (Figure 4c; Figure S20, Supporting Information), resulting in the positively charged state of the Co site. Specifically, Co─N_3_/EG exhibited a charge transfer of 0.89 eV, higher than that of Co─N_3_/G (0.72 eV), indicating that the Co oxidation state was higher in Co─N_3_/EG, in consistency with the experimental results.

To understand the difference in the HER activity between Co─N_3_/EG and Co─N_3_/G, the Gibbs free energy for the adsorption of the H^*^ (Δ*G*_H*_) was calculated (Figure 4d). The Δ*G*_H*_ value of Co─N_3_/EG (0.1 eV) was much closer to the thermoneutral point than that of Co─N_3_/G (−1.50 eV), suggesting that the Co sites in Co─N_3_/EG had significantly decreased hydrogen adsorption strength and thus improved HER activity. Then, the H^*^ adsorption configuration on the active site was explored (Figure S21, Supporting Information). It was found that the H^*^ preferentially occupied the top position of the Co center in Co─N_3_/G, while H^*^ adsorbed on the side position of the Co center in Co─N_3_/EG. Based on the principle of the orbital symmetry match in Valence Bond Theory, the *d_z_* ^2^ orbital with perpendicular orientation was typically deemed as the optimal choice to enable the maximum electronic coupling with the adsorbed H^*^. However, the moderate (neither too strong nor too weak) interaction between the active site and the intermediate was the desirable condition according to the Sabatier principle. With these aspects in mind, the PDOS of the *s* orbital of H^*^ and the *d* orbital of Co were investigated (Figure 4e,f). The results showed that there were large overlaps between the H‐*s* orbital and the Co‐*d_z_* ^2^ orbital in Co─N_3_/G, suggesting its strong Co−H interaction. In comparison, for Co─N_3_/EG, the H‐*s* orbital interacted with the *d_xy_* orbital of Co atom, and the weakened Co−H interaction was indicated by the significantly reduced electronic coupling because of the moderate orbital alignment capability. Figure 4g displayed the surficial electron density difference and schematic diagram for the orbital interaction between H^*^ and Co with different orientations in Co─N_3_/EG and Co─N_3_/G. The quantitative results suggested that 0.13 electrons were donated from Co atom to H^*^ in Co─N_3_/G for the symmetrical orbitals between H‐*s* and Co‐*d_z_* ^2^, whereas the value was reduced to 0.04 for Co─N_3_/EG with the H‐*s* and Co‐*d_xy_* interaction, highlighting the weakened H^*^ adsorption on the Co sites in Co─N_3_/EG for improved HER activity.

## 3 Conclusion

In summary, we demonstrated that the introduction of vacancy defects neighboring the single‐atom Co─N_3_ sites via NaHCO_3_ etching can promote the HER catalysis via regulated adsorption configuration of the reaction intermediates. Structural analysis confirmed the modified electronic structure of Co sites in Co─N_3_/EG for its variations in the oxidation state and the spin state of the Co center. Impressively, the optimized Co─N_3_/EG achieved an exceptional overpotential of 78 mV at 10 mA cm^−2^ and a small Tafel slope of 45.2 mV dec^−1^, making it one of the best catalysts among earth‐abundant SACs. DFT calculations indicated that the vacancy defect induced the hydrogen intermediate preferentially to adsorb on the side position of the atomic Co sites, which resulted in moderated orbital symmetry match and weakened the binding strength of intermediates, thereby resulting in improved HER activity. This study introduced an efficient approach to modulating the electronic structure and catalytic reactivity of M─N─C catalysts and provided valuable insights into the structure‐property relationship at the atomic level for designing high‐performance SACs.

## Conflict of Interest

The authors declare no conflict of interest.

## Supporting information



Supporting Information

## Data Availability

The data that support the findings of this study are available from the corresponding author upon reasonable request.
