# Supplementary material for: Boosting the Hydrogen Evolution Activity of a Low‐Coordinated Co─N─C Catalyst via Vacancy Defect‐Mediated Alteration of the Intermediate Adsorption Configuration
Source: Adv Sci (Weinh). 2025 Jan 13;12(9):2415665. doi: 10.1002/advs.202415665 (PMC11884577; doi:10.1002/advs.202415665)
Supplement: Supplementary file 1 — Supporting Information [file ADVS-12-2415665-s001.pdf]

ADVANCED SCIENCE
Open Access

Supporting Information

for *Adv. Sci.*, DOI 10.1002/advs.202415665

Boosting the Hydrogen Evolution Activity of a Low-Coordinated Co—N—C Catalyst via Vacancy Defect-Mediated Alteration of the Intermediate Adsorption Configuration

Qianwei Song, Zhichao Gong, Jianbin Liu, Kang Huang, Gonglan Ye, Shuwen Niu and Huilong Fei**

Supporting Information for

Boosting the Hydrogen Evolution Activity of a Low-Coordinated Co−N−C Catalyst via Vacancy Defect-Mediated Alteration of the Intermediate Adsorption Configuration

Qianwei Song, Zhichao Gong, Jianbin Liu, Kang Huang, Gonglan Ye, Shuwen Niu[*], *Huilong Fei*[*]

Q. Song, Z. Gong, J. Liu, K. Huang, G. Ye, H. Fei

State Key Laboratory for Chemo/Biosensing and Chemometrics, Advanced Catalytic Engineering Research Center of the Ministry of Education and College of Chemistry and Chemical Engineering, Hunan University, Changsha, 410082, P. R. China.

S. Niu

College of Chemistry and Chemical Engineering Institution, Qingdao University, Qingdao, 266071, P. R. China.

*Correspondence: niusw@qdu.edu.cn; hlfei@hnu.edu.cn

Methods

Materials. All reagents were obtained from commercial sources and were used without further purification. Cobalt chloride hexahydrate ($CoCl_2{\cdot}6H_2O$, > 99.9%) was obtained from Aladdin Reagent. Sodium bicarbonate ($NaHCO_3$), potassium bicarbonate ($KHCO_3$) and sodium carbonate (Na_2CO_3) were purchased from Shanghai Titan Scientific Co., Ltd. Nafion 117 solution (5 wt%) was purchased from Sigma-Aldrich. Concentrated sulfuric acid (H_2SO_4), phosphoric acid (H_3PO_4), hydrochloric acid (HCl), and potassium permanganate ($KMnO_4$) were bought from Sinopharm Chemical Reagent Co., Ltd. The natural graphite flakes were purchased from Nanjing Xianfeng Nano Material Technology Co., Ltd. Graphene oxide (GO) was synthesized by the oxidation of natural graphite flakes (50 mesh) with a modified Hummers method. The ultrapure water was used in the experiments with a resistivity of 18.25 MΩ cm^{-1}.

Synthesis of Co−N_3/EG. An aqueous dispersion containing GO (5 mL, 2 mg mL^{-1}) and $CoCl_2{\cdot}6H_2O$ (100 μL, 3 mg mL^{-1}) was prepared by sonicating for 30 min and stirring for 60 min. Subsequently, controlled amounts of $NaHCO_3$ (mass proportion of 8.4% relative to GO) were added into the dispersion, followed by the addition of H_2O (15 mL) to dilute the dispersion. The as-prepared dispersion was freeze-dried to avoid significant restacking of GO sheets. The dried sample was then placed in the center of a quartz tube furnace. After purging the system with Ar/NH_3 (150/50 sccm) for 30 min, the temperature of the furnace was ramped at 2 °C min^{-1} up to 200 °C and then at 25 °C min^{-1} to 750 °C. The two annealing steps, including a slow ramping step (2 °C min^{-1}) and fast ramping step (25 °C min^{-1}) were employed to slowly release CO_2 by

$NaHCO_3$ decomposition and the construction of Co−N active sites at high temperature, respectively. The reaction was allowed to proceed for 1 h and the blackish powder was collected after the natural cooling of the furnace. Finally, the powder was acid-leached in 0.5 M H_2SO_4 for 24 h to remove the residual Na_2CO_3 or Na_2O and underwent a secondary annealing treatment at 750 °C for 30 min in Ar to obtain the final product of Co−N_3/EG. For the control samples of N/EG and Co−N_3/G, the preparation procedure was similar to that of Co−N_3/EG except that the addition of cobalt salt or $NaHCO_3$ was omitted in the precursor solution, respectively.

Characterizations

SEM was performed on Regulus 8100 with 10 kV working voltage. Aberration-corrected STEM characterization was conducted on a ThermoFisher Themis Z microscope equipped with two aberration correctors under 300 kV. High-angle annular dark field (HAADF)-STEM images were recorded using a convergence semiangle of 25 mrad and inner and outer collection angles of 41 and 200 mrad, respectively. EDS was carried out using 4 in-column Super-X detectors. XRD characterizations were determined on a Bruker D8 Advance diffractometer with Cu Kα radiation ($\lambda \approx 1.54$ Å) at a scan rate of 10° min^{-1}. XPS was collected on Thermo Scientific K-Alpha by Al Kα radiation and the elemental spectra were all corrected with respect to C1*s* peak at 284.8 eV. Raman spectra were collected on a Thermo Scientific DXR Raman microscope with a 532 nm laser. The metal loadings were determined by ICP-MS (Agilent 7900). Hard X-ray absorption spectra at Co *K*-edge were obtained at BL1W1B

beamline of Beijing Synchrotron Radiation Facility in fluorescence mode using a Si (111) double-crystal monochromator.

Electrochemical Measurements

The electrochemical measurements were carried out in a standard three-electrode setup with 0.5 M H_2SO_4 as electrolyte using a CHI 760E workstation. Graphite rod and Hg/Hg_2SO_4, K_2SO_4(sat.) were used as the counter electrode and reference electrode, respectively. All of the potentials were quoted against a reversible hydrogen electrode (RHE) according to $E_{\mathrm{RHE}} = E_{\mathrm{Hg/Hg_2SO_4}} + 0.704$ V based on the calibration results (Figure S9) and all potentials were corrected with 95% *iR* compensation. To prepare the working electrode, 1 mg catalyst was dispersed in 250 μL of ethanol along with 20 μL of 5 wt% Nafion solution, followed by bath sonication for 30 min to achieve a homogeneous suspension (~4 mg mL^{-1}). 14 μL catalyst ink was loaded onto a 5 mm diameter glassy carbon electrode. Linear sweep voltammograms (LSV) with the rate of 5 mV s^{-1} were performed to evaluate the HER activity. Electrochemical impedance spectroscopy (EIS) measurements were carried out at −0.15 V (*vs*. RHE) with an amplitude of 5 mV over a frequency range from 100 kHz to 0.01 Hz. Stability was evaluated by cycling the catalysts at a scan rate of 50 mV s^{-1} in the potential range of −0.1 to 0 V (*vs*. RHE) for 1000, 2000 and 4000 cycles and additionally by chronopotentiometric test operated at the constant current density of 10 mA cm^{-2} for 40 h.

Calculation of the effective moments (μ_{eff}) and the unpaired electrons (*n*)

The molar susceptibility was calculated by the following equation:

$$\chi_m = \frac{MM_{Co}}{Hm_{Co}}$$

where M was the measured magnetic moment during ZFC, H was the magnetic field intensity, $M_{Co} = 58.93$ mol g^{-1} was the atomic weight of Co and m_{Co} was the mass of Co determined from ICP-MS.

The effective moment (μ_{eff}, the calculated molecular moments) was fitted based on the Langevin equation at the high temperature region of M-T curves, where the magnetic ordering was entirely broken by thermal disturbance:

$$\chi_m = \chi_0 + \frac{N_A \mu_{eff}^2 \mu_0}{3kT}$$

where χ_m and χ_0 were the molar susceptibility and molar diamagnetic susceptibility, respectively. The latter was generally considered as a constant at different temperatures. $N_A = 6.02\times10^{23}$ was the Avogadro constant, $\mu_0 = 4\pi\times10^{-7}$ N A^{-2} was the vacuum permeability, $k = 1.38\times10^{-23}$ J K^{-1} was the Boltzmann constant and T was temperature.

Calculation of turnover frequency (TOF)

The TOF value was determined based on the previous report,[1] and the details were presented as follows:

$$\text{TOF}(\text{H}_2/\text{s}) = \frac{\text{\# Total hydrogen turnovers per geometric area}}{\text{\# Active sites per geometric area}}$$

The number of total hydrogen turnovers was calculated from the current density extracted from the LSV polarization curve according to the following equation:

$$\text{Total hydrogen turnovers} = (|j|\frac{\text{mA}}{\text{cm}^2})(\frac{1\ \text{C/s}}{1000\ \text{mA}})(\frac{1\ \text{mol e}^-}{96485\ \text{C}})(\frac{1\ \text{mol}}{2\ \text{mol e}^-})(\frac{6.022\times10^{23}\ \text{H}_2}{1\ \text{mol H}_2})$$

$$=3.12\times10^{15}\frac{1\ \text{H}_2/\text{s}}{\text{cm}^2}\ \text{per}\ \frac{\text{mA}}{\text{cm}^2}$$

The number of active sites in the Co−N_3/EG catalyst was calculated from the mass loading on the glassy carbon electrode, the Co contents and the Co atomic weight, assuming each Co center accounted for one active site:

$$\text{Number of active sites}=\left(\frac{\text{catalyst loading per geometric area}(x\ \text{g/cm}^2)\times\text{Co wt\%}}{\text{Co M}_\text{w}\ (\text{g/mol})}\right)\left(\frac{6.022\times10^{23}\ \text{Co atoms}}{1\ \text{mol Co}}\right)$$

$$=\left(\frac{0.285\times10^{-3}\ \text{g/cm}^2\times1.33\ \text{wt\%}}{58.93\ \text{g/mol}}\right)\left(\frac{6.022\times10^{23}\ \text{Co atoms}}{1\ \text{mol Co}}\right)$$

$$=6.57\times10^{16}\ \text{Co sites per cm}^2$$

Finally, the current density from the LSV polarization curve can be converted into TOF values according to:

$$\text{TOF}=\left(\frac{3.12\times10^{15}}{6.57\times10^{16}}\times|\,j\,|\right)=0.047\times|\,j\,|$$

For Co−N_3/EG, the current density at the η of 100 mV was measured to be 35.53 mA cm^{-2}, which corresponded to TOF of 1.67 s^{-1}. TOF values for other sample were calculated by the same methodology.

Estimation of the electrochemical active surface area (ECSA)

The ECSA of different catalysts was calculated from the mass-normalized double-layer capacitance (C_{dl}) according to equation below:

$$\text{ECSA}=\frac{C_{dl}}{C_s}$$

where C_{dl} was the measured double-layer capacitance from the CVs at the scan rates of 20, 40, 60, 80, 100 mV s^{-1} in the non-Faradaic region. The C_s was the specific capacitance of a flat standard electrode with 1 cm^2 of real surface area. For our estimates of surface area, we used an averaged specific capacitance of C_s=40 μF cm^{-2} based on typical reported values.[2]

Computational Details

All the spin-polarized density functional theory (DFT) calculations were conducted using the projected augmented wave (PAW) method implemented in the Vienna Ab initio Simulation Package.[3] The exchange-correlation potential was modeled with the Perdew-Burke-Ernzerhof (PBE) functional within the generalized gradient approximation (GGA). To avoid the interactions between neighboring slabs, a vacuum layer of 15 Å was applied along the z-axis. The DFT dispersion correction (DFT-D_3) method was used to treat the van der Waals interactions. For geometry optimization, the kinetic energy cutoff was set to 500 eV and the self-consistent field tolerance was 1.0×10^{-5} eV. The Brillouin zone was sampled by 3*3*1 and 6*6*1 Monkhorst–Pack mesh k-point for surface calculation and the density of states (DOS) calculation, respectively. All atoms were fully relaxed until the force reaching the convergence threshold of 0.02 eV $Å^{-1}$. The free energy changes (ΔG_{H*}) for H* adsorption on Co−N_3/G and Co−N_3/EG surfaces were calculated according to the equation: $\Delta G_{H*} = E_{sur\text{-}H} - E_{sur} - 1/2E_{H_2} + \Delta E_{ZPE} - T\Delta S$, where $E_{sur\text{-}H}$ was the total energy of surface covered with a H, E_{sur} was the energy of clean surface, and E_{H_2} was the energy of H_2 in the gas phase, ΔE_{ZPE} was the zero-point energy change and ΔS was the entropy change. For this study, the value of $\Delta E_{ZPE} - T\Delta S$ on catalyst surface was 0.24 eV for H adsorption.[4]

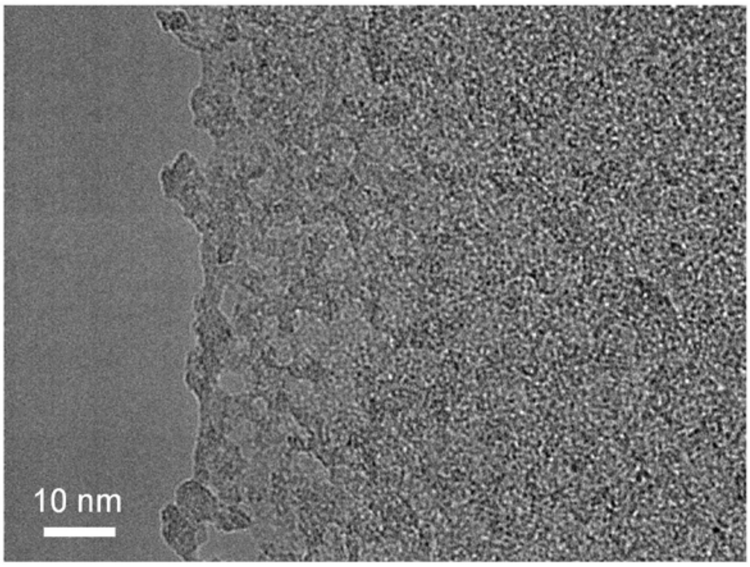

141 **Figure S1.** TEM image of N/EG.

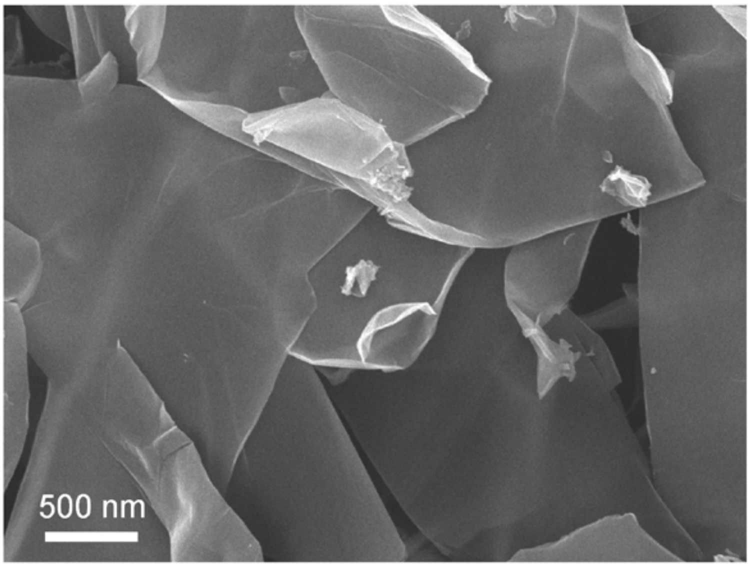

Figure S2. SEM image of Co−N_3/G.

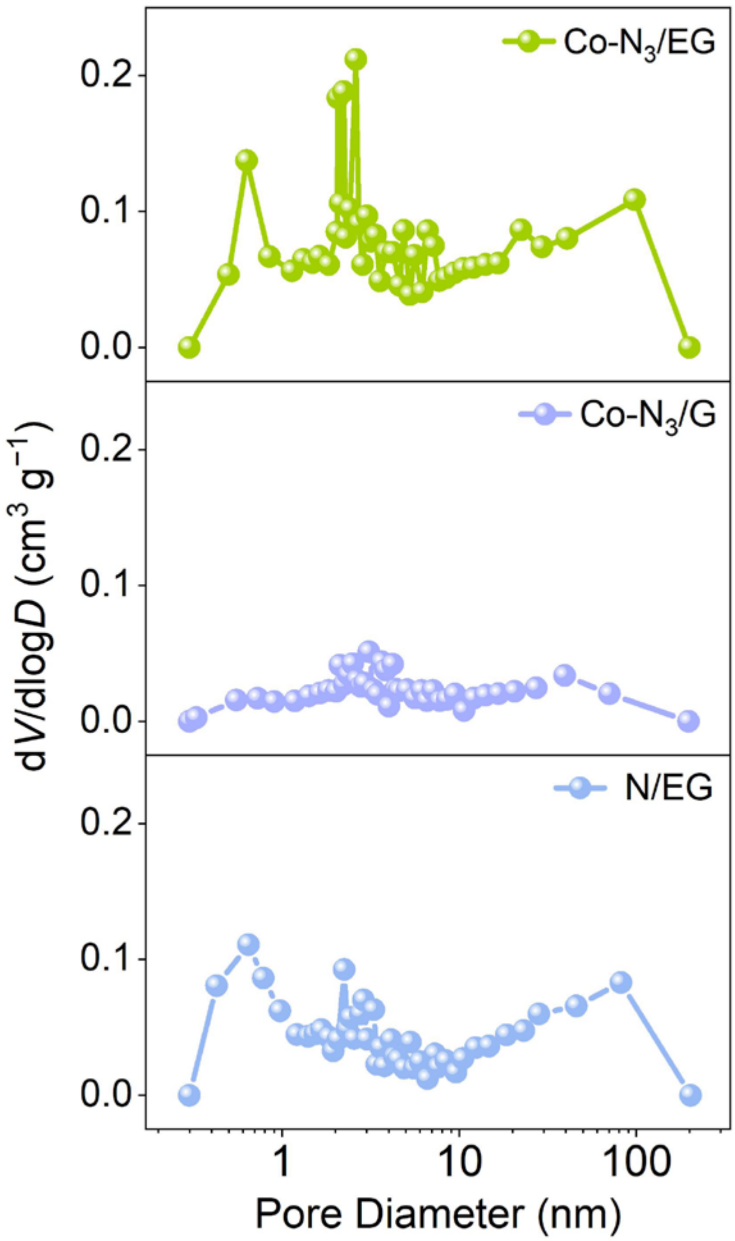

Figure S3. Pore size distribution curves of Co−N3/EG, Co−N3/G and N/EG.

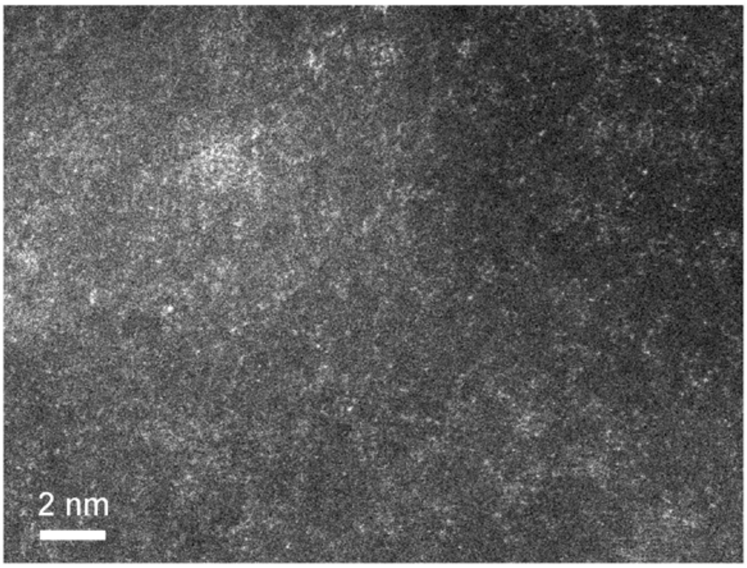

Figure S4. Aberration-corrected HAADF-STEM image of Co−N_3/G.

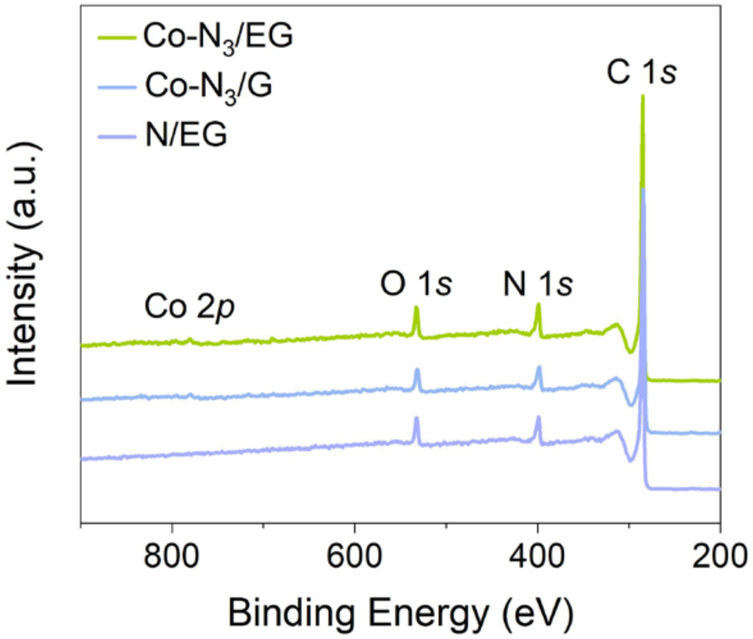

Figure S5. XPS survey spectra of Co−N_3/EG, Co−N_3/G and N/EG.

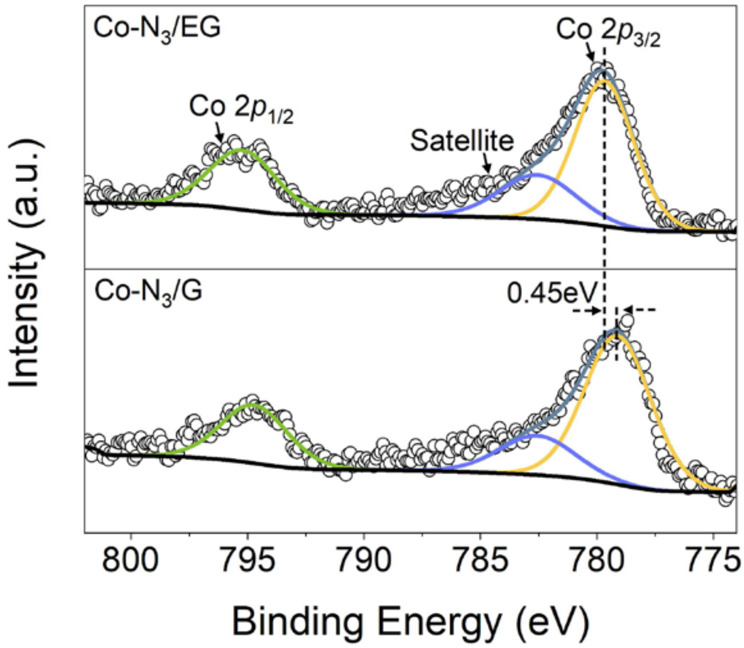

Figure S6. High-resolution XPS Co 2*p* spectra of Co−N_3/EG and Co−N_3/G.

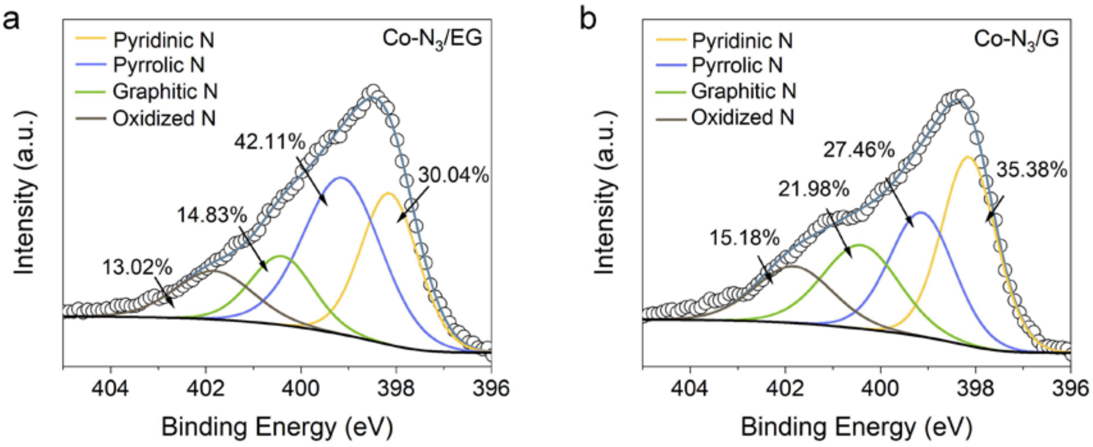

Figure S7. High-resolution XPS N 1*s* spectra of a) Co−N_3/EG and b) Co−N_3/G.

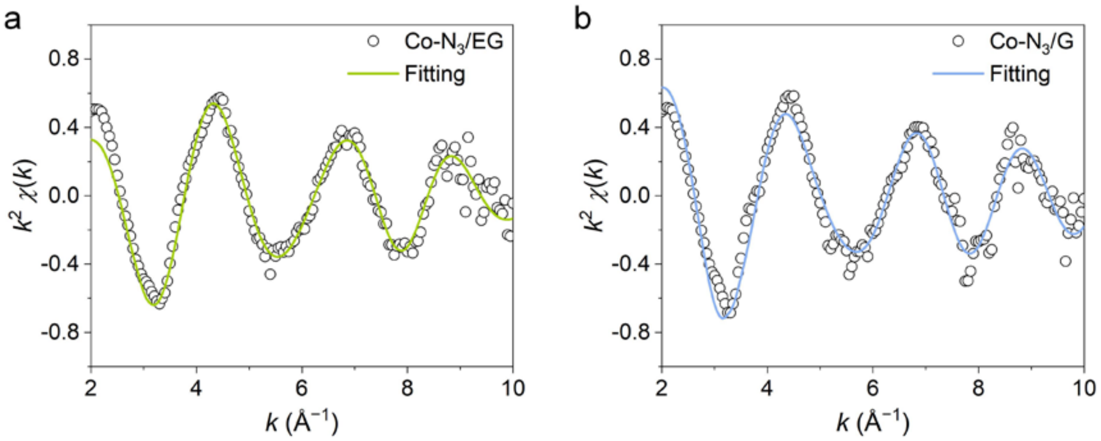

154

155 **Figure S8.** EXAFS fitting curves in *k*-space of a) Co−N_3/EG and b) Co−N_3/G.

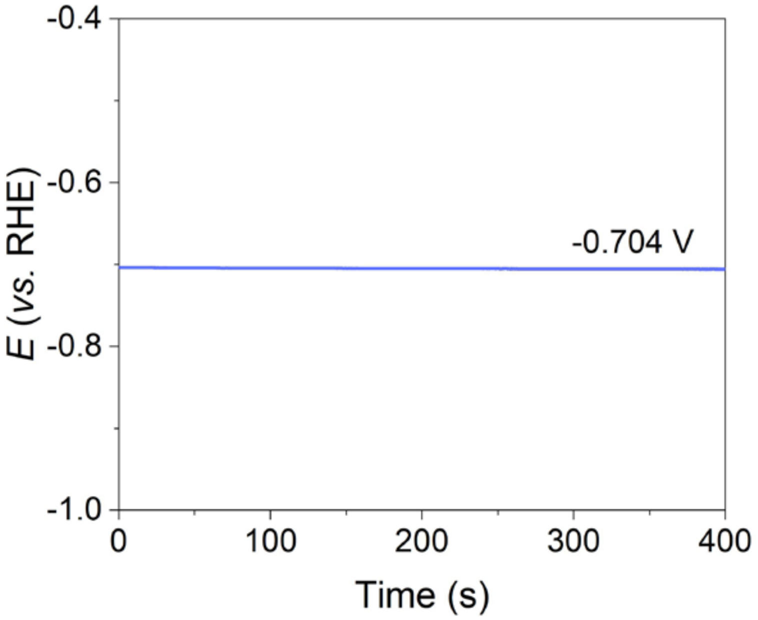

Figure S9. Calibration of Hg/Hg_2SO_4, K_2SO_4 (sat.) reference electrode in 0.5 M H_2SO_4.

Potential calibration of the reference electrode was conducted using a standard three-electrode system with Pt as working/counter electrode and Hg/Hg_2SO_4, K_2SO_4 (sat.) as the reference electrode. During calibration, the open circuit potential (OCP) was monitored over time in H_2-saturated 0.5 M H_2SO_4 until a stable OCP value was attained, which was considered as the calibrated potential of the reference electrode.

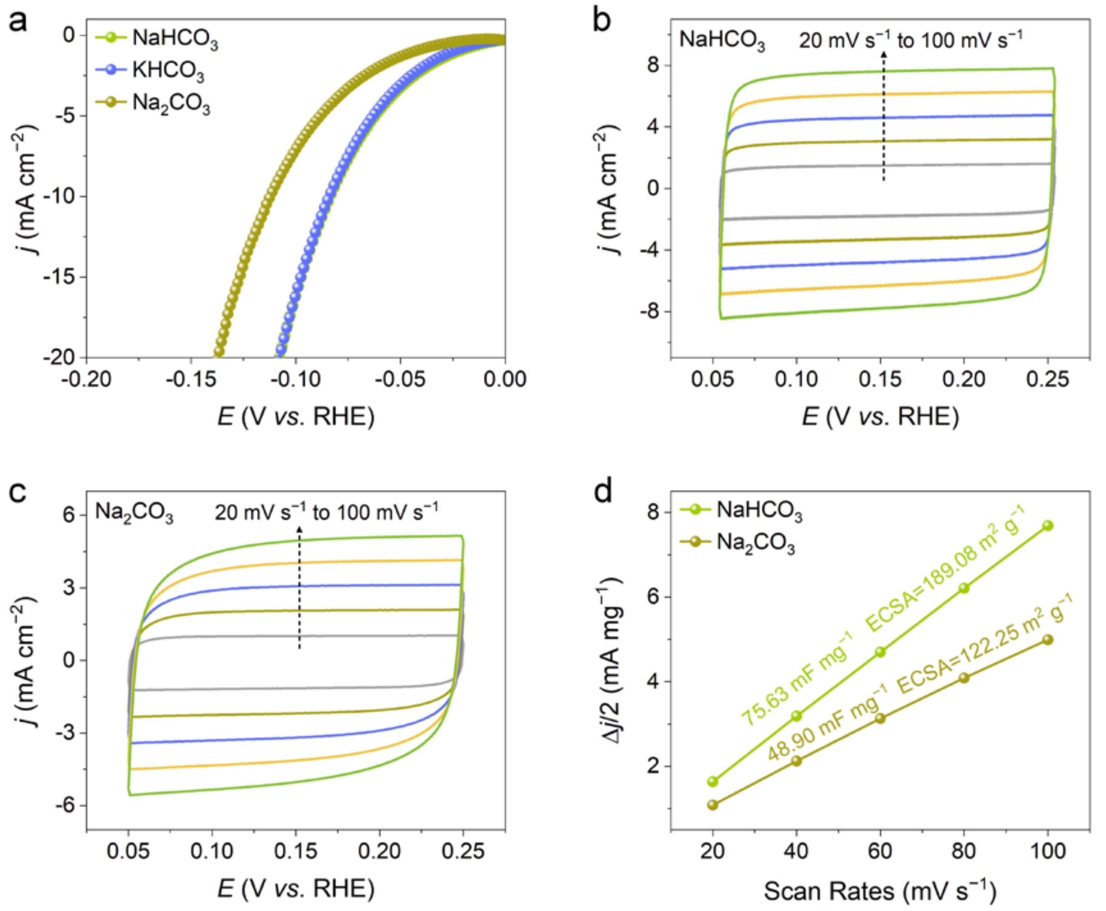

Figure S10. a) The LSV plots of the samples prepared with different carbonate species. CV curves of samples prepared with the addition of b) $NaHCO_3$ and c) Na_2CO_3 in a non-Faradaic region from 0.05 V to 0.25 V *vs*. RHE at scan rates of 20 mV s^{-1}, 40 mV s^{-1}, 60 mV s^{-1}, 80 mV s^{-1} and 100 mV s^{-1}. d) The corresponding capacitive currents as a function of scan rates. Because of the lower decomposition temperature of $NaHCO_3$ compared to Na_2CO_3, it could release more CO_2, which led to higher ECSA and thus better catalytic activity of the prepared sample.

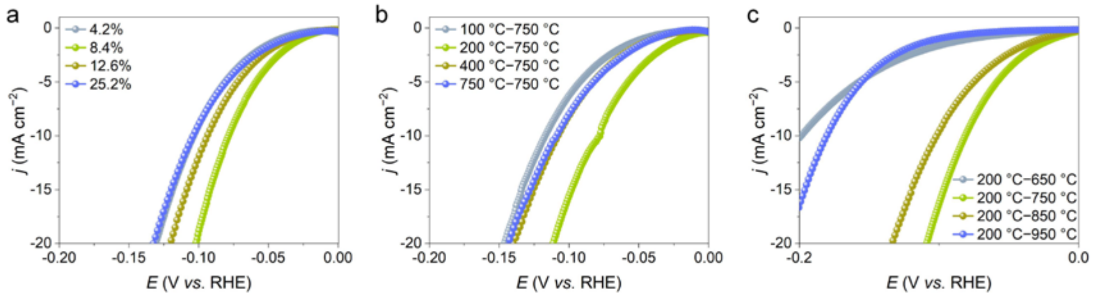

Figure S11. a) The LSV plots of the samples prepared with different $NaHCO_3$ amounts (mass proportion relative to GO precursor). It can be seen that the HER activity increased first and then decreased along with the increased addition of $NaHCO_3$. The optimal sample was prepared with 8.4% $NaHCO_3$. The LSV plots of the samples prepared with b) different first-step and c) different second-step annealing temperatures. The samples were labeled by the target temperature of each step. The relationship of calcination temperature and HER performance showed a volcanic trend and the sample annealed at 200 °C and 750 °C in the first and second annealing step exhibited the best HER performance.

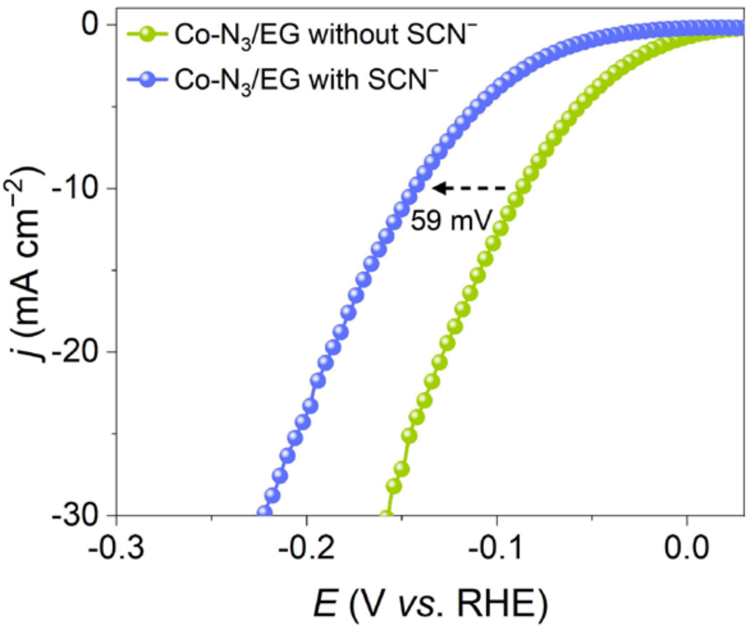

Figure S12. LSV curves of Co−N_3/EG tested in 0.5 M H_2SO_4 before and after KSCN addition. Prior to the measurement of LSV, the electrode was dipped in 0.5 M H_2SO_4 containing 50 mM KSCN for 4 h to fully poison the Co active sites. The η increased by 59 mV to deliver a current density of 10 mA cm^{-2} after KSCN addition.

186 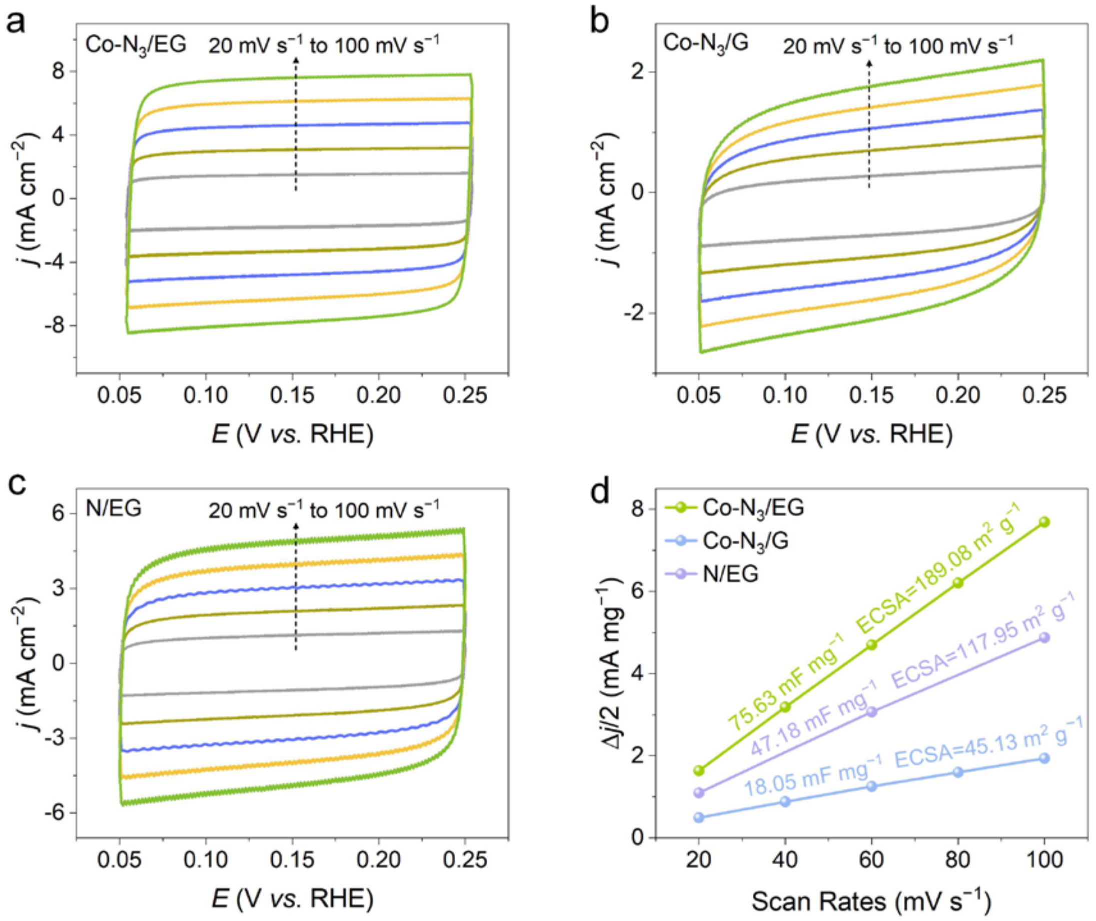

187 **Figure S13.** CV curves of a) Co−N3/EG, b) Co−N3/G and c) N/EG in a non-Faradaic
188 region from 0.05 V to 0.25 V *vs.* RHE at scan rates of 20 mV s^{-1}, 40 mV s^{-1}, 60 mV s
189 $^{-1}$, 80 mV s^{-1} and 100 mV s^{-1}. d) The capacitive currents as a function of scan rates for
190 Co−N3/EG, Co−N3/G and N/EG.

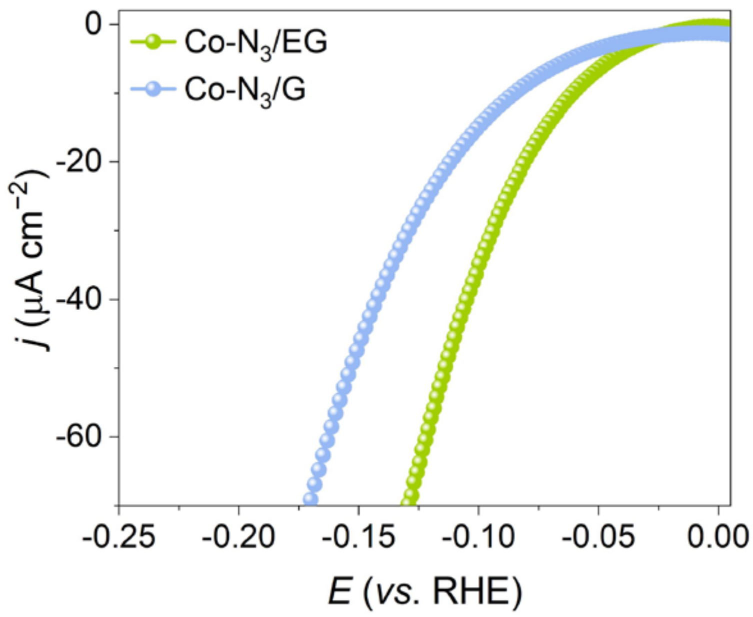

Figure S14. ECSA-normalized LSV curves of Co−N_3/EG and Co−N_3/G.

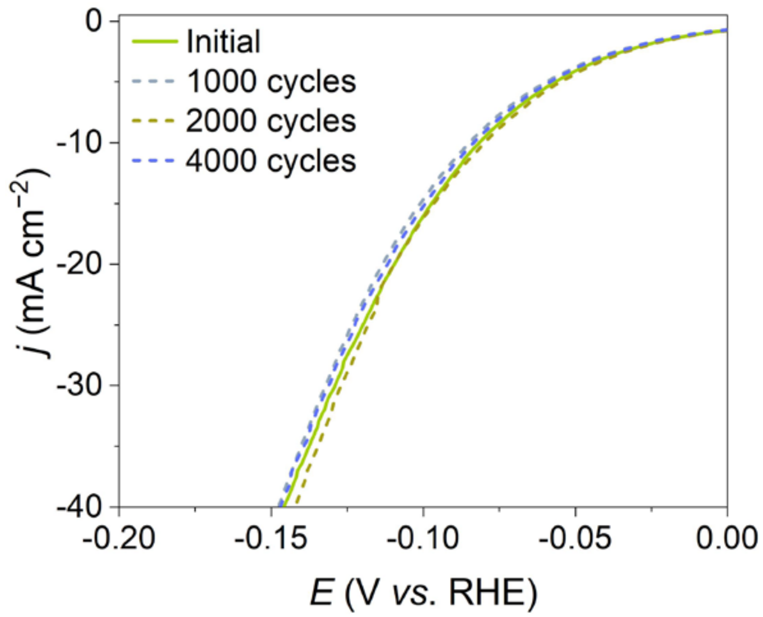

Figure S15. Cycling stability test of Co−N_3/EG. LSV plots of Co−N_3/EG were collected initially and after 1000, 2000 and 4000 CV cycles from −0.1 to 0 V (*vs.* RHE) at the scan rate of 50 mV s^{-1}.

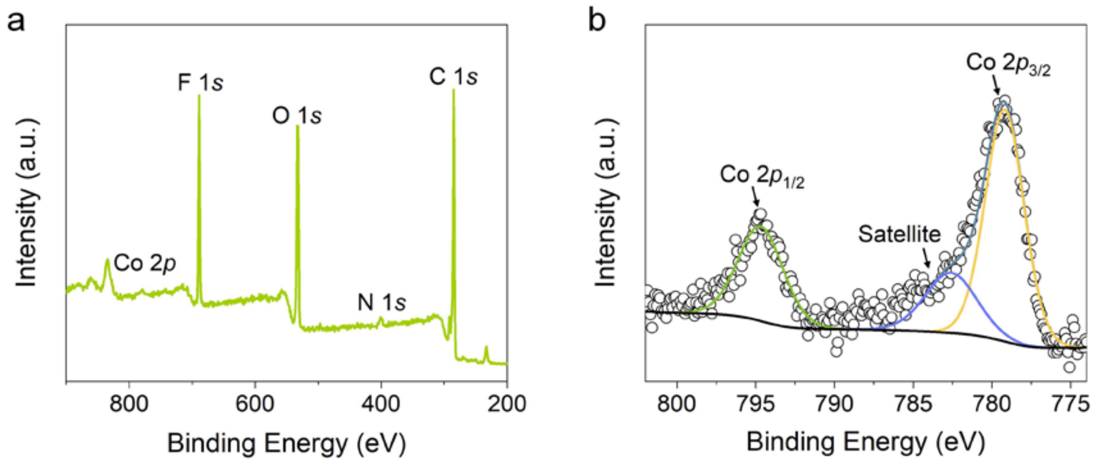

Figure S16. a) XPS survey spectrum and b) high-resolution XPS Co 2*p* spectrum of Co−N_3/EG after long-term stability test. XPS survey spectrum showed the presence of C, N, O, Co elements and the additional peak for F arose from the Nafion binder added during electrode preparation. The high-resolution Co 2*p* XPS spectrum suggested the ionic state of Co species.

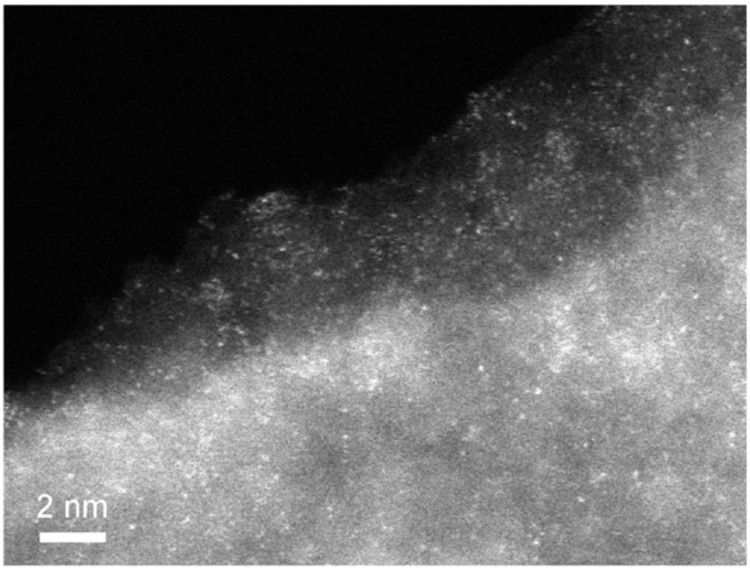

Figure S17. HAADF-STEM image of Co−N_3/EG after long-term stability test.

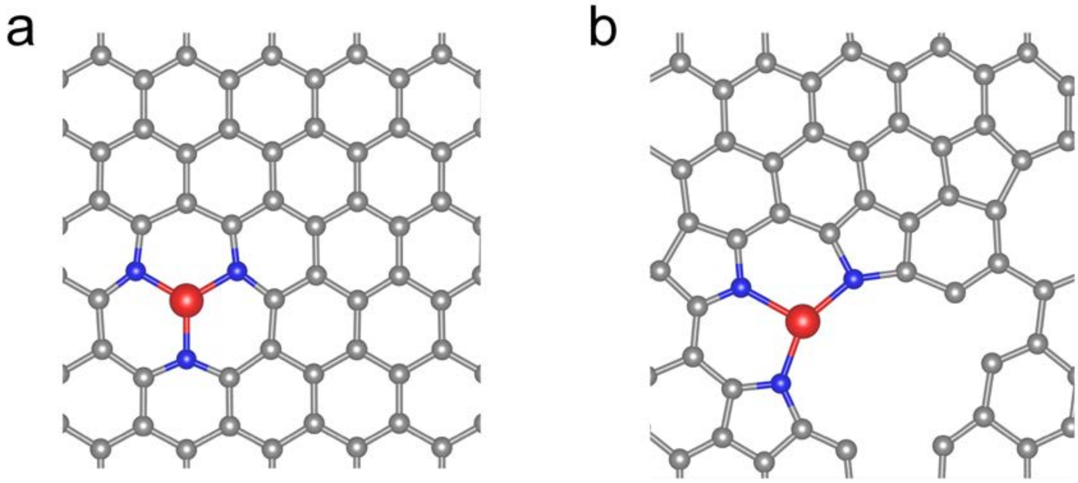

Figure S18. The calculation models of a) Co−N_3/G and b) Co−N_3/EG surfaces.

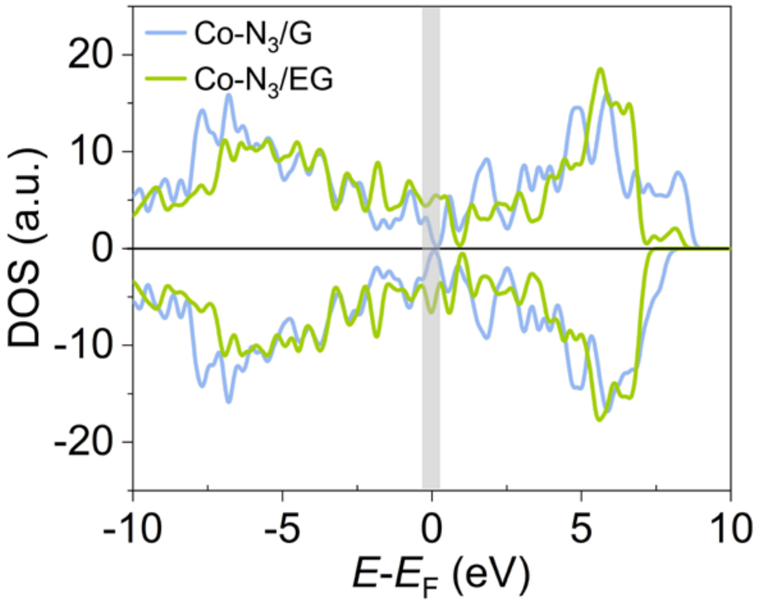

207

208 **Figure S19.** The calculated total DOS of Co−N_3/G and Co−N_3/EG.

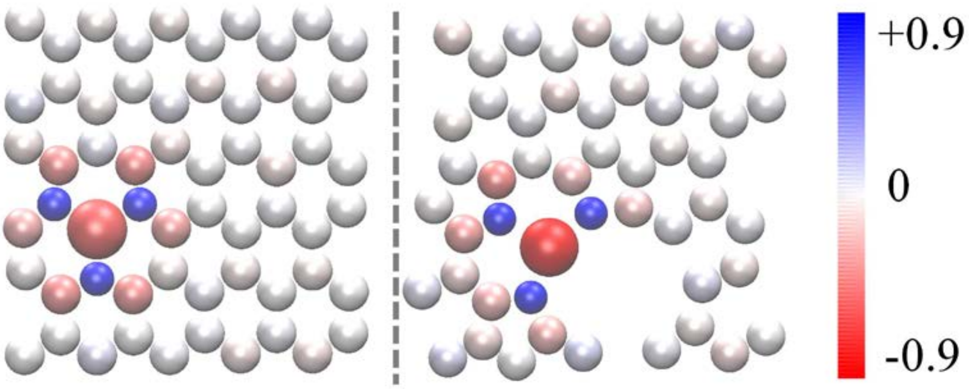

Figure S20. Bader charge of Co−N_3/G (left) and Co−N_3/EG (right) surfaces.

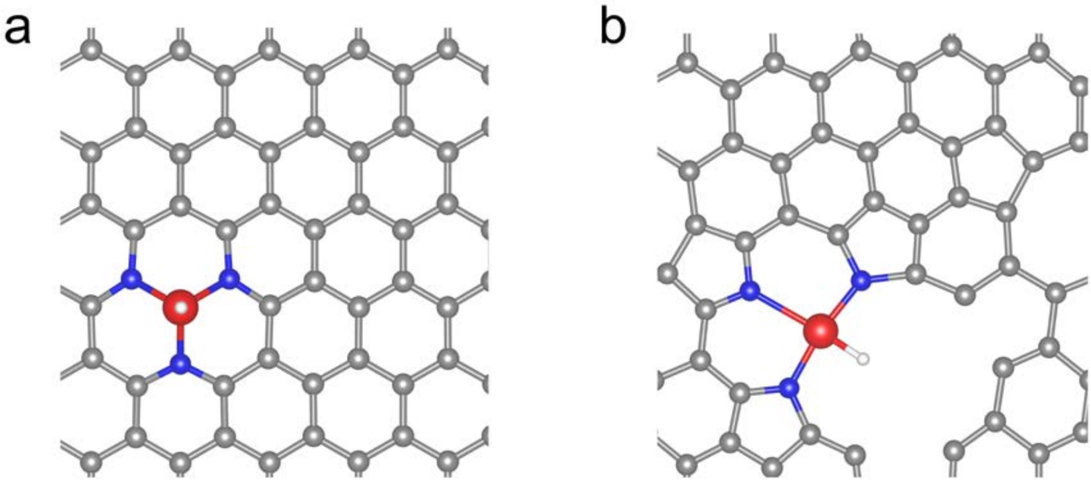

Figure S21. The calculated structural configurations for H* adsorption on a) Co−N_3/G and b) Co−N_3/EG surfaces.

Table S1. Summary of the compositions in Co−N_3/EG, Co−N_3/G and N/EG. The C, N, and O contents were determined by XPS, while the Co content was determined by ICP-MS.

Catalysts	C(at%)	N(at%)	O(at%)	Co(wt%)
Co−N_3/EG	86.73	7.39	5.62	1.33
Co−N_3/G	86.94	7.28	5.48	1.30
N/EG	86.38	7.98	5.63	/

Table S2. Fitting parameters[a] of Co *K*-edge EXAFS curves.

Catalysts	Path	*N*	*R* (Å)	σ^2(Å^2)	ΔE_0 (eV)	*R*, %
Co foil[b]	Co-Co	12	2.49	0.008	7.1	0.006
CoO[c]	Co-O	2.8	1.87	0.004	-7.1	0.002
Co−N_3/EG[d]	Co-N	2.8	1.87	0.004	-7.1	0.002
Co−N_3/G[e]	Co-N	3.0	1.91	0.003	1.5	0.012

[a] *N*, coordination number; *R*, distance between absorber and backscatter atoms; σ^2, Debye-Waller factor to account for both thermal and structural disorders; ΔE_0, inner potential correction; *R*-factor indicates the goodness of the fit. Error bounds (accuracies) that characterize the structural parameters obtained by EXAFS spectroscopy were estimated as N ± 20%; R ± 1%; σ^2 ± 20%; ΔE_0 ± 20%. S_0^2 was fixed to 0.85 as determined from Co foil fitting. Bold numbers indicate fixed *N* according to the crystal structure. [b] Fitting range: 2.5 ≤ *k* (/Å) ≤ 10.0 and 1.0 ≤ *R* (Å) ≤ 3.0. [c] Fitting range: 2.5 ≤ *k* (/Å) ≤ 10.0 and 1.0 ≤ *R* (Å) ≤ 2.4. [d] Fitting range: 2.5 ≤ *k* (/Å) ≤ 10.0 and 1.0 ≤ *R* (Å) ≤ 2.4. [e] Fitting range: 2.5 ≤ *k* (/Å) ≤ 10.0 and 1.0 ≤ *R* (Å) ≤ 2.4.

228 **Table S3.** Comparison of the electrocatalytic HER activity of Co−N_3/EG with other
229 recently reported Co-based SACs in 0.5 M H_2SO_4 solution.

Catalysts	Metal content (wt%)	Catalyst loading (mg cm^{-2})	η_{10} (mV)	Tafel slope (mV dec^{-1})	TOF (s^{-1}) @η=100 mV	Ref.
Co-N_3/EG	1.33	0.285	78	45.2	1.67	This work
Co-N_3/G	1.30	0.285	177	76.5	0.80	This work
Co-P_1N_3	1.6	0.102	98	47	1.60	[5]
CoN_3-CSG	2.10	0.285	82	59	0.8	[6]
Co-I-N/G	2.45	0.285	52	56.1	1.88	[7]
CoN_x/C	0.14	2	133	57	0.39	[8]
Co-SAS/HOPNC	0.49	0.6	137	52	0.41	[9]
Co-NG	2.48	0.285	147	82	0.1	[1]
Co-NG-MW	1.10	0.1	175	80	0.38	[10]
CoNG-Cl	4.1	0.35	130	41	/	[11]
Co_1/PCN	0.3	0.5	151	74	/	[12]
Co-SAs/PTF-600	0.85	0.204	94	50	/	[13]
CNT/Co-PcC	1.4	0.8	200	80	/	[14]
Co/NCNT/NG	3.7	0.24	123	67	/	[15]
SACo-N/C	2.16	1	169	118	/	[16]
Co-NG-5010-10	2.4	0.302	146	65	/	[17]
Co SA MoO_3	2.4	0.8	112	98	/	[18]
Co-C-N	0.19	/	138	55	/	[19]

Reference

[1] H. L. Fei, J. C. Dong, M. J. Arellano-Jiménez, G. L. Ye, N. D. Kim, E. L. G. Samuel, Z. W. Peng, Z. Zhu, F. Qin, J. M. Bao, M. J. Yacaman, P. M. Ajayan, D. L. Chen, J. M. Tour, *Nat. Commun.* **2015**, *6*, 8668.

[2] M. Yan, Z. Wei, Z. Gong, B. Johannessen, G. Ye, G. He, J. Liu, S. Zhao, C. Cui, H. Fei, *Nat. Commun.* **2023**, *14*, 368.

[3] a) G. Kresse, J. Furthmüller, *Phys. Rev. B* **1996**, *54*, 11169; b) Y. Yao, S. Hu, W. Chen, Z.-Q. Huang, W. Wei, T. Yao, R. Liu, K. Zang, X. Wang, G. Wu, W. Yuan, T. Yuan, B. Zhu, W. Liu, Z. Li, D. He, Z. Xue, Y. Wang, X. Zheng, J. Dong, C.-R. Chang, Y. Chen, X. Hong, J. Luo, S. Wei, W.-X. Li, P. Strasser, Y. Wu, Y. Li, *Nat. Catal.* **2019**, *2*, 304.

[4] J. K. Nørskov, T. Bligaard, A. Logadottir, J. R. Kitchin, J. G. Chen, S. Pandelov, U. Stimming, *J. Electrochem. Soc.* **2005**, *152*, J23.

[5] J. Wan, Z. Zhao, H. Shang, B. Peng, W. Chen, J. Pei, L. Zheng, J. Dong, R. Cao, R. Sarangi, Z. Jiang, D. Zhou, Z. Zhuang, J. Zhang, D. Wang, Y. Li, *J. Am. Chem. Soc.* **2020**, *142*, 8431.

[6] K. Huang, Z. Wei, J. Liu, Z. Gong, J. Liu, M. Yan, G. He, H. Gong, Y. Hu, Y. He, S. Zhao, G. Ye, H. Fei, *Small* **2022**, *18*, 2201139.

[7] J. Liu, D. Wang, K. Huang, J. Dong, J. Liao, S. Dai, X. Tang, M. Yan, H. Gong, J. Liu, Z. Gong, R. Liu, C. Cui, G. Ye, X. Zou, H. Fei, *ACS Nano* **2021**, *15*, 18125.

[8] H.-W. Liang, S. Brüller, R. Dong, J. Zhang, X. Feng, K. Müllen, *Nat. Commun.*

2015, *6*, 7992.

[9] T. Sun, S. Zhao, W. Chen, D. Zhai, J. Dong, Y. Wang, S. Zhang, A. Han, L. Gu, R. Yu, X. Wen, H. Ren, L. Xu, C. Chen, Q. Peng, D. Wang, Y. Li, *Proc. Natl. Acad. Sci.* **2018**, *115*, 12692.

[10] H. Fei, J. Dong, C. Wan, Z. Zhao, X. Xu, Z. Lin, Y. Wang, H. Liu, K. Zang, J. Luo, S. Zhao, W. Hu, W. Yan, I. Shakir, Y. Huang, X. Duan, *Adv. Mater.* **2018**, *30*, 1802146.

[11] G. Wan, C. Yang, W. Zhao, Q. Li, N. Wang, T. Li, H. Zhou, H. Chen, J. Shi, *Adv. Mater.* **2017**, *29*, 1703436.

[12] L. Cao, Q. Luo, W. Liu, Y. Lin, X. Liu, Y. Cao, W. Zhang, Y. Wu, J. Yang, T. Yao, S. Wei, *Nat. Catal.* **2018**, *2*, 134.

[13] J.-D. Yi, R. Xu, G.-L. Chai, T. Zhang, K. Zang, B. Nan, H. Lin, Y.-L. Liang, J. Lv, J. Luo, R. Si, Y.-B. Huang, R. Cao, *J. Mater. Chem. A* **2019**, *7*, 1252.

[14] Y. J. Sa, S. O. Park, G. Y. Jung, T. J. Shin, H. Y. Jeong, S. K. Kwak, S. H. Joo, *ACS Catal.* **2018**, *9*, 83.

[15] L. Yang, Y. Lv, D. Cao, *J. Mater. Chem. A* **2018**, *6*, 3926.

[16] Y. Wang, L. Chen, Z. Mao, L. Peng, R. Xiang, X. Tang, J. Deng, Z. Wei, Q. Liao, *Sci. Bull.* **2019**, *64*, 1095.

[17] Y. Zhang, W. Li, L. Lu, W. Song, C. Wang, L. Zhou, J. Liu, Y. Chen, H. Jin, Y. Zhang, *Electrochim. Acta* **2018**, *265*, 497.

[18] K. Kim, C. Kim, S.-M. Bak, C.-Y. Nam, J. H. Moon, *Chem. Eng. J* **2024**, *488*, 150976.

[19] Z.-L. Wang, X.-F. Hao, Z. Jiang, X.-P. Sun, D. Xu, J. Wang, H.-X. Zhong, F.-L. Meng, X.-B. Zhang, *J. Am. Chem. Soc.* **2015**, *137*, 15070.
